# Network reprogramming after resection of occipital meningioangiomatosis: Evidence from multimodal localization and longitudinal fMRI

**DOI:** 10.1016/j.ebr.2026.100874

**Published:** 2026-05-19

**Authors:** Changquan Wang, Bin Chen, Jing Hong, Jiwen Xu

**Affiliations:** a Department of Functional Neurosurgery, Comprehensive Epilepsy Unit, Ruijin Hospital Luwan Branch, Shanghai Jiao Tong University, School of Medicine, 49 South Chongqing Road, Shanghai, China; b Department of Neurosurgery, Clinical Neuroscience Center, Comprehensive Epilepsy Unit, Ruijin Hospital, Shanghai Jiao Tong University School of Medicine, 197 Ruijin Er Road, Shanghai, China

**Keywords:** Meningioangiomatosis, Occipital lobe epilepsy, PET, Resting-state fMRI, Network reorganization

## Abstract

**Purpose:**

To characterize the epileptic network and postoperative network remodeling in a rare case of right cuneus meningioangiomatosis (MA) with electro-clinical discordance, we integrated electroencephalography (EEG), fluorodeoxyglucose positron emission tomography (FDG-PET), and resting-state fMRI (rs-fMRI).

**Methods:**

A 16-year-old male with drug-resistant focal impaired-awareness seizures and a right cuneus lesion underwent preoperative multimodal evaluation. Preoperative video-EEG, magnetic resonance imaging (MRI)/computed tomography (CT), and FDG-PET were obtained; gross total resection confirmed MA. Pre- and postoperative rs-fMRI were processed in DPABI. We computed lesion-seed functional connectivity (FC), fractional amplitude of low-frequency fluctuation (fALFF), Regional homogeneity (ReHo), and weighted degree centrality (DC; *r* > 0.25), then generated post–pre difference maps using Fisher z-transformed data and retained the top 5% of absolute voxel changes (cluster size >50 voxels).

**Results:**

Ictal EEG showed onset in the right parieto-occipital region with rapid spread to right temporal and frontal areas. FDG-PET revealed focal hypometabolism in the right cuneus and remote hypometabolism in the right mesial temporal and left central regions. Postoperatively, lesion-based FC decreased in bilateral primary sensorimotor, early visual, and superior temporal/opercular cortices, but increased in the ipsilesional dorsal occipito-parietal network. fALFF and ReHo decreased widely in peri-Rolandic and occipital regions; fALFF and DC increased in medial/superior frontal cortex and bilateral precuneus; DC decreased in inferior frontal opercular/triangular areas.

**Conclusion:**

Multimodal findings localized the seizure onset to the right posterior region with widespread propagation. Postoperative rs-fMRI showed network changes consistent with partial normalization, paralleling sustained seizure freedom—though causality cannot be inferred from a single case.

## Highlights

• A rare sporadic meningioangiomatosis in the right cuneus is reported.
• Multimodal presurgical evaluation resolved the electro-clinical discordance.
• Post-resection findings support seizure control via large-scale network reprogramming.

## 1 Introduction

Meningioangiomatosis (MA) is a rare, benign, hamartomatous lesion of the leptomeninges and cerebral cortex characterized by perivascular proliferation of meningothelial and fibroblast-like cells forming plaque-like structures within cortex, first described by Bassoe in 1915 [1]. Most lesions arise in the temporal or frontal lobes; posterior cortex (parietal/occipital) involvement is uncommon, with fewer than 10 occipital cases reported in the literature [2]. Epilepsy is the most frequent clinical manifestation, occurring in up to 82% of symptomatic cases [2], [3]. Seizure semiology often reflects lesion location; however, rapid network propagation can produce electro-clinical discordance and complicate localization.

We report a rare case of sporadic MA in the right cuneus causing drug-resistant epilepsy with discordant electro-clinical features, where electroencephalography (EEG) and fluorodeoxyglucose positron emission tomography (FDG-PET) suggested fronto–temporal involvement despite a posterior lesion. We integrate long-term video-EEG (VEEG), structural magnetic resonance imaging (MRI)/ computed tomography (CT), fluorodeoxyglucose positron emission tomography (FDG-PET) and pre/postoperative resting-state fMRI (rs-fMRI) to localize the epileptogenic network and to characterize postoperative network remodeling. This case expands the sparse literature on posterior MA and illustrates a network-based approach to focal epilepsy.

## 2 Case presentation

A right-handed, full-term 16-year-old male with normal development developed seizures two years earlier. Typical events began abruptly without aura and consisted of staring and impaired awareness, eye blinking, prominent left-hand fumbling automatisms progressing at times to bilateral manual automatisms, and frequent coughing/drooling. Seizure frequency persisted at approximately weekly despite valproate (1250 mg/day) and levetiracetam (1000 mg/day). Cognitive function was normal (MoCA 30/30; WAIS 99), with no anxiety, depression, or psychotic symptoms.

## 3 Presurgical Evaluation

### 3.1 Long-term video-EEG

Electrodes were placed according to the international 10–20 system using a 32-channel configuration. Recording parameters were set as follows: A sampling rate of 1024 Hz, a high-pass filter at 0.5 Hz, and a low-pass filter at 70 Hz

**Interictal EEG:** Epileptiform discharges predominated over bilateral temporal and occipital regions; after medication reduction, paroxysms increased maximally over the right temporal/occipital regions. Additional slow-wave activity appeared over left fronto–centro–parietal regions with bilateral temporal sharp waves (right-predominant).

**Fig. 1:**
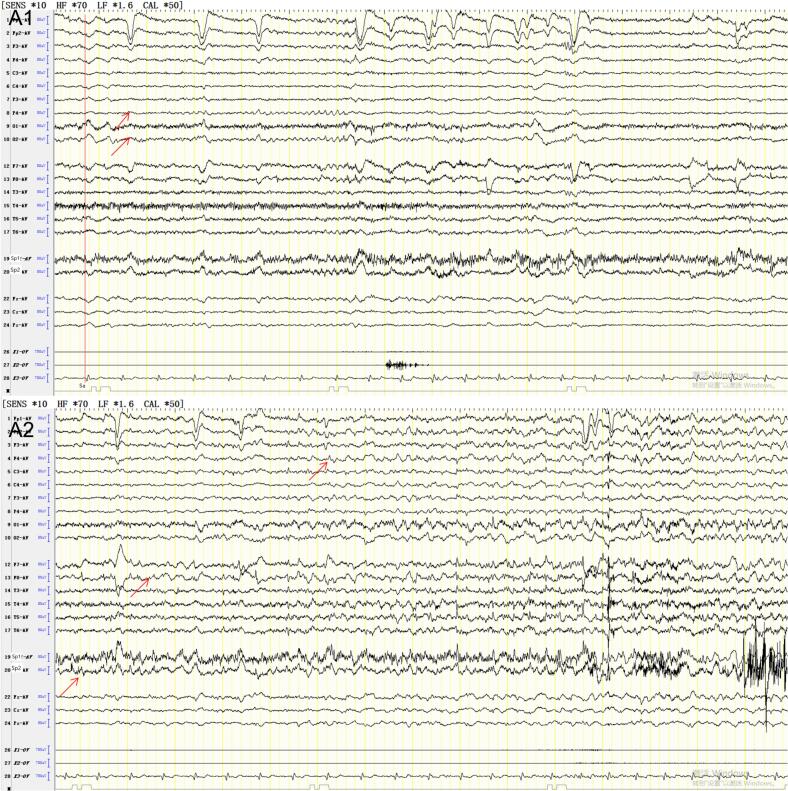
Scalp electroencephalography (EEG) during clinical seizure (two consecutive frames). (A1) Ictal EEG onset demonstrates low-amplitude sharp waves in the right parietal-occipital region (arrow, channels P4/O2). (A2) Rapid anterior propagation with involvement of the right sphenoidal electrode and right frontotemporal leads (arrow; Sp2/F8), with further spread to P4.

**Ictal EEG (Clinical Seizure):** Onset with rhythmic, low-amplitude sharp activity in the right parietal–occipital region. Rapid anterior propagation evolved into rhythmic low-to-medium-amplitude sharp–slow complexes in right sphenoidal, temporal, frontopolar, and frontal regions, eventually bilateral/diffuse. (Fig. 1).

**Electrographic seizures** (*n* = 3; all with coughing: occurring once during a seizure and twice at its termination). Onsets commonly emphasized left frontal/central/parietal channels with low-amplitude slow waves, followed by bilateral temporal spike recruitment (right-predominant), then spread to frontal/central/temporal regions and diffuse involvement (∼70 s).

The detailed temporal evolution of the electrographic activity is provided in Supplementary Fig. 1.

### 3.2 Structural and metabolic imaging

Neuroimaging acquisition: The MR study included T1 magnetization-prepared rapid acquisition gradient echo (voxel size 0.75 × 0.75 × 0.76 mm), T2 sequences (voxel size 0.63× 0.63 × 1 mm), T2-Fluid-attenuated inversion recovery (FLAIR) sequences (voxel size 0.8 × 0.8 × 0.8 mm), using a 3.0-T scanner (uMR 890, United Imaging Healthcare, Shanghai, China) with a dedicated 64-channel head coil. Patients were required to fast and abstain from sugary beverages for a minimum of six hours prior to the procedure. Following intravenous administration of 18F-FDG (0.1 mCi/kg), subjects were instructed to rest in a quiet, dimly lit environment for 30 min before proceeding to standardized imaging protocol acquisition.

**Fig. 2:**
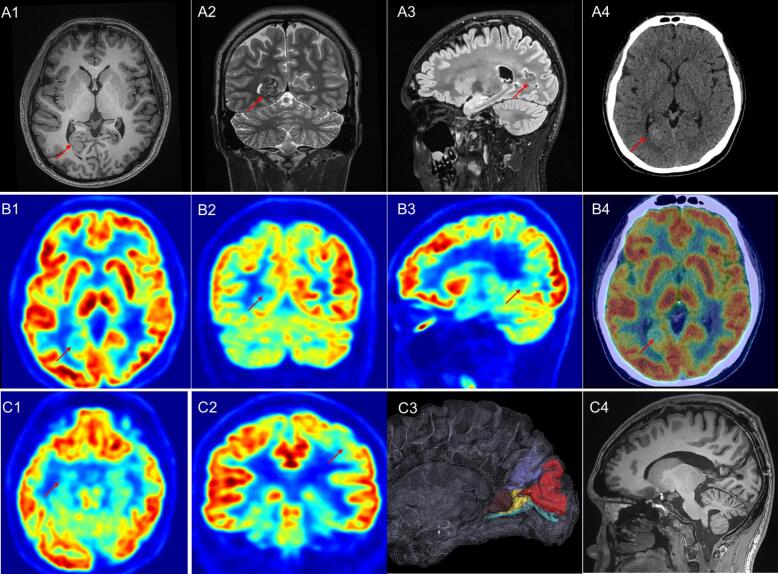
Structural neuroimaging and metabolic imaging. (A1-A4) Structural neuroimaging. (A1) Axial T1-weighted MRI shows cortical thickening with iso- to hypointense signal in the right cuneus (arrow). (A2) Coronal T2-weighted MRI demonstrates mixed iso- to hypointense signal within the lesion (arrow). (A3) Sagittal FLAIR MRI shows mixed signal abnormality (arrow). (A4) Axial CT revealed a focal hyperdensity consistent with calcification. (B1-B4). Lesion co-localization on FDG-PET. (B1) Axial, (B2) coronal, and (B3) sagittal FDG-PET images demonstrate focal hypometabolism in the right cuneus (red arrows), co-localizing with the structural lesion. (B4) Axial CT–PET fusion provides anatomical reference. (C1–C4) Remote metabolic involvement and postoperative outcome. (C1) Axial FDG-PET shows right mesial temporal hypometabolism (arrow). (C2) Axial FDG-PET shows left central hypometabolism (arrow). (C3) 3D surface reconstruction (Slicer/Freesurfer) depicts the anatomical context of the lesion: right cuneus (red), ventral posterior cingulate (brown), calcarine sulcus (blue), parieto-occipital sulcus (purple). The manually delineated lesion is shown in yellow. (C4) Postoperative (3-month) T1-weighted MRI confirms complete resection of the lesion. Abbreviations: CT, computed tomography. FDG-PET, Fluorodeoxyglucose positron emission tomography, MRI, magnetic resonance imaging

**MRI/CT:** A lesion (1.8 × 1.8 × 2.0 cm) centered in the right cuneus with extensions toward the parieto–occipital sulcus, calcarine fissure, and ventral posterior cingulate cortex. The lesion showed cortical thickening with iso- to hypointense T1 and mixed iso- to hypointense T2/FLAIR signal; CT revealed a focal hyperdensity consistent with calcification. No significant mass effect was observed (Fig. 2).

**FDG-PET:** Focal hypometabolism precisely co-localized to the right cuneus lesion. Additional relative hypometabolism was observed in right temporal structures and the left central region (Fig. 2).

## 4 Surgery and histopathology

The patient underwent tailored right occipital craniotomy with gross total resection. Histopathology confirmed MA involving leptomeninges and cortex, with bland spindle-cell perivascular proliferation partitioning cortex into islands, vascular hyalinization, calcification with psammoma bodies, and degenerative atrophy of entrapped neurons. Immunohistochemistry: CD34(+), ATRX(+), H3K27me3(+), p53 (focal weak+), p16 (rare+); negative for GFAP, Olig2, Nestin, neuronal markers (neurofilament, synaptophysin, chromogranin), calretinin, IDH1, PGM 1, BRAF V600E, S100, and EMA.

## 5 Postoperative course

The postoperative course was uneventful. The patient remained seizure-free at 9-month follow-up (Engel Class I). Medications unchanged. Postoperative EEG at 3 months (Supplementary Fig. 2) showed no epileptiform activity, consistent with the sustained clinical remission.

## 6 Resting-state fMRI

### 6.1 Imaging and methods

Postoperative rs-fMRI was acquired 3 months after resection. Anti-seizure medications were unchanged between the pre- and postoperative imaging sessions. Single-shot echo-planar BOLD fMRI: TR 700 ms, TE 30 ms, flip angle 52°, 63 contiguous slices (2.5 mm), 2.5 mm isotropic voxels, 700 volumes (∼8 min). Two sessions (pre- and post-resection) were processed in Data Processing & Analysis for Brain Imaging (DPABI) [4], a MATLAB-based open-source toolbox designed for resting-state fMRI data analysis: discard first 10 volumes; slice-timing correction; realignment; co-registration to structural image; gray matter (GM)/white matter (WM)/cerebrospinal fluid (CSF) segmentation; DARTEL normalization to MNI152; nuisance regression (motion, WM, CSF); normalization to 3 mm voxels; band-pass 0.01–0.1 Hz. ALFF/fALFF were computed per DPABI defaults. Resting-state fMRI analyses included: (1) lesion-seed functional connectivity (FC), computed using a manually delineated lesion region of interest (ROI) on structural MRI based on visible lesion boundaries to quantify temporal correlations between the lesion and all other brain voxels; (2) fractional amplitude of low-frequency fluctuations (fALFF), reflecting spontaneous low-frequency signal power; (3) regional homogeneity (ReHo), measuring local functional synchronization; and (4) weighted degree centrality (DC; *r* > 0.25), quantifying whole-brain functional connectedness. Post − Pre difference maps were computed on Fisher z–transformed images. In this single-case design, descriptive thresholds retained the top 5% absolute voxel differences with clusters >50 voxels.

### 6.2 Results (post − pre)

**Lesion–seed FC:** decreased in bilateral precentral/postcentral gyri, supplementary motor area (SMA)/paracentral lobules, mid-cingulate, early visual cortex (calcarine, lingual, cuneus), cerebellar lobules IV–VI, and superior temporal/opercular regions (including right Rolandic operculum/insula); increased in ipsilesional dorsal occipito-parietal network (right cuneus, superior/middle occipital, precuneus, angular, superior parietal), left superior parietal, and right middle frontal gyrus.

**fALFF:** decreased in sensorimotor and occipital cortices (including right postcentral); increased in medial/superior frontal cortex, bilateral precuneus, cerebellar Crus I/II, and smaller areas of middle/inferior temporal and orbitofrontal cortex.

**ReHo:** decreased in peri-Rolandic, SMA/paracentral, mid-cingulate, and occipital (calcarine/lingual/cuneus) cortices, and right postcentral gyrus; increased in medial superior frontal cortex, bilateral precuneus, cerebellar Crus I/II, and right orbitofrontal cortex.

**DC (weighted):** decreased in bilateral inferior frontal opercular/triangular regions and right inferolateral occipital (lingual/inferior occipital) cortex; increased in medial/superior frontal cortex (superior frontal gyrus, medial prefrontal/SMA), bilateral precuneus/posterior cingulate, and cerebellar Crus I/II.

**Fig. 3:**
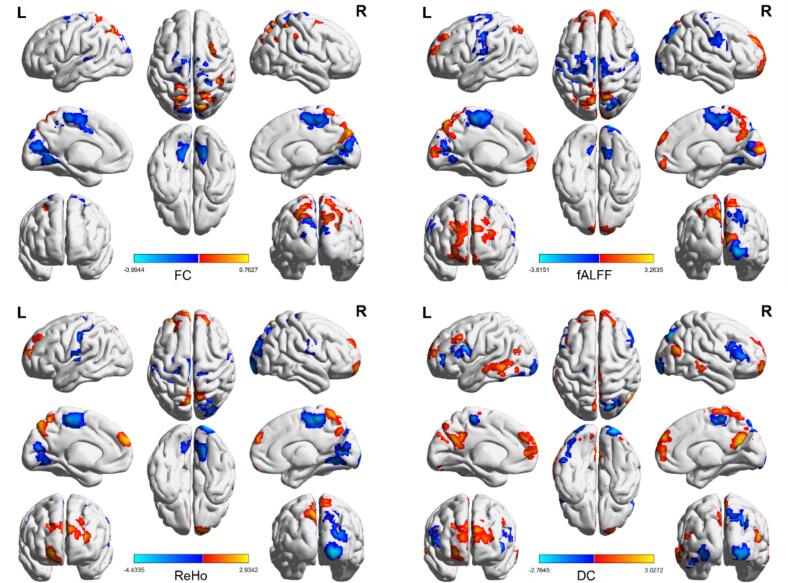
Postoperative functional brain network reorganization after right cuneus resection. Maps show pre- to postoperative rs-fMRI differences (Post–Pre), thresholded at top 5% absolute voxel-wise change (cluster >50). Warm colors (red/yellow) indicate increases; cool colors (blue) indicate decreases. Seed-to-voxel FC: Reduced lesion coupling with primary sensorimotor/early visual systems; increased integration in dorsal occipito-parietal/precuneus regions. fALFF: Decreased amplitude in primary sensorimotor/visual cortices; increased signal in medial frontal and precuneus hubs. ReHo: Reduced local synchrony in primary systems; increased synchrony in medial prefrontal/precuneus, suggesting shift toward default-mode activity. DC: Increased centrality in DMN and medial frontal hubs; decreased centrality in inferior frontal opercular nodes linked to propagation. (For interpretation of the references to colour in this figure legend, the reader is referred to the web version of this article.)

Postoperative rs-fMRI showed expected remodeling (Fig. 3): reduced activity in primary sensorimotor and early visual cortices, weaker coupling with superior temporal/opercular regions (dampened operculo-insular/temporal propagation), and greater hub strength in precuneus and medial/superior frontal cortex (default mode network (DMN)/dorsal parietal normalization).

## 7 Discussion

This sporadic MA case in the right cuneus goes beyond simple clinico-pathological correlation, illustrating focal epilepsy as a disorder of large-scale brain networks. The progression from paradoxical semiology to curative surgery and mechanistic validation exemplifies a network-based approach to understanding and treating epilepsy.

### 7.1 Electro-clinical paradox explained by network propagation

The diagnostic challenge in this case was the apparent “temporal-like” semiology (staring with impaired awareness, manual automatisms, coughing/drooling) despite converging electrophysiological and imaging evidence for a posterior onset [5]. This electro-clinical discordance is explained by an epileptogenic network mechanism [6]: a right cuneus lesion adjacent to the calcarine fissure and parieto-occipital sulcus, extending into the ventral posterior cingulate, facilitates rapid propagation via established occipital white-matter pathways [7], [8]. Impaired awareness and manual automatisms are commonly linked to temporal–limbic involvement, yet they are not localizing to the ictal onset zone and instead reflect early recruitment of temporal networks following posterior onset. Coughing and drooling indicate engagement of opercular/insular and autonomic pathways, secondarily activated during rapid propagation [9], [10]. Consistent with this interpretation, ictal EEG localized the onset to the right parieto-occipital region, confirmed by focal hypometabolism in the right cuneus on FDG-PET. FDG-PET also revealed remote hypometabolism in the right temporal lobe and left central region—evidence of broader network involvement that explains both the semiology and the EEG evolution: right posterior onset followed by rapid anterior/temporal spread and downstream left fronto-central involvement.

### 7.2 Network reprogramming after lesion resection

Focal resection inevitably alters local rs-fMRI signals due to tissue removal, perfusion changes, and cessation of epileptiform activity. In this case, however, postoperative alterations were not confined to the perilesional region, but involved distributed network nodes previously implicated by electroclinical and metabolic abnormalities, with convergent findings across multiple rs-fMRI metrics. This pattern was consistent with coordinated postoperative network reorganization [11], including reduced sensorimotor/visual activity and propagation-related connectivity, together with relatively enhanced default-mode and dorsal parietal hubs. Imaging was performed 3 months postoperatively after resolution of acute effects and anti-seizure medication remained unchanged, supporting this interpretation. Overall, this case suggests that a focal epileptogenic lesion can affect large-scale functional networks and that seizure freedom after resection may reflect coordinated network reorganization. This pattern may extend to other lesional focal epilepsies such as focal cortical dysplasia and low-grade tumors, where a single pathological node anchors the network [12]. Larger, controlled longitudinal studies across etiologies and lesion locations are needed to determine whether such network reprogramming consistently accompanies successful epilepsy surgery.

### 7.3 Multimodal integration: EEG–PET– Rs-fMRI correspondence

Epileptogenic focus: MRI/CT revealed a calcified right cuneus lesion, consistent with focal hypometabolism on PET. Postoperative rs-fMRI showed reduced lesion-seed FC and amplitude (fALFF/ReHo) in adjacent occipital regions, indicating local down-regulation after lesion removal. EEG showed ictal spread from occipital to temporal/opercular/frontal regions via ventral and dorsal pathways [8], matching postoperative FC reductions in superior temporal/opercular cortices and decreased DC in inferior frontal opercular/triangular areas, indicating suppressed operculo-insular/temporal propagation. PET hypometabolism in right temporal and left central regions aligned with rs-fMRI changes in sensorimotor networks (peri-Rolandic fALFF/ReHo/FC decreases), while increased DC and fALFF in precuneus and medial frontal cortex suggested restored DMN/dorsal parietal function following decoupling from the posterior lesion.

Clinical outcome: Complete seizure freedom (Engel I) after lesionectomy indicates that a single posterior lesion sustained a distributed epileptic network. Resecting the primary lesion silenced remote symptomatogenic zones without extended resections [13].

7.4 Pathological specificity and imaging–pathology correlation

Leptomeningeal and cortical involvement, perivascular spindle cell proliferation, and vasculocentric calcification are pathognomonic for MA [2], [14], [15]. The immunohistochemical profile further supports this interpretation and refines the differential diagnosis [16]. Specifically, strong CD34 immunoreactivity is a hallmark of the spindle cells in MA; preserved ATRX and H3K27me3 expression argues against infiltrative glial neoplasms; negative EMA staining makes meningioma less likely; and absence of IDH1 R132H and BRAF V600E immunoreactivity effectively excludes common diffuse gliomas and low-grade glioneuronal tumors. Taken together, the integrated histomorphological and immunophenotypic findings strongly favor MA over other cortical or leptomeningeal tumor-associated lesions.

The pathology also revealed structural substrates with established relevance to epileptogenicity. Cortical involvement, neuronal islanding, focal cortical atrophy, and calcified abnormalities collectively indicate chronic local network disruption [15]. These histopathological features aligned closely with the neuroimaging phenotype—particularly the CT-detectable calcifications and the T2-hypointense core—supporting the interpretation that the radiological abnormalities reflect the underlying calcified and fibrovascular architectural pathology [2], [17], [18].

### 7.4 Clinical implications and future directions

Clinically, this case shows that longitudinal rs-fMRI can complement structural imaging and electroclinical follow-up by depicting postoperative reorganization at the network level [11]. The patient remains seizure-free 9 months postoperatively with no change to the antiseizure medication regimen. Medication taper will be considered only after 2 years of sustained seizure freedom, guided by standard clinical criteria—especially absence of clinically meaningful interictal epileptiform discharges on follow-up EEG, consistent with common postoperative practice [19]. Prospective, controlled studies in lesional focal epilepsy are needed to determine whether multi-metric reprogramming correlates with seizure freedom, cognitive recovery, and antiseizure medication reduction.

### 7.5 Limitations

This study has several limitations. First, the single-case design—lacking healthy or surgical controls with other epileptogenic etiologies—precludes statistical inference and limits generalizability; thus, our imaging analyses are descriptive, using top-5% difference maps (cluster threshold >50 voxels) with no *p*-values reported. Second, although rs-fMRI captures functional network organization, it remains vulnerable to physiological noise, head motion, and neurovascular coupling effects, all of which may vary with age and clinical state.

## 8 Conclusion

This case suggests that the clinical phenotype of lesional epilepsy may be influenced by the functional network context of the lesion. Post-resection rs-fMRI demonstrated coordinated network changes temporally associated with seizure freedom; however, these descriptive observations do not establish causality. Further studies with appropriate control groups are needed.

## Declaration of generative AI in scientific writing.

During the preparation of this work we used DeepSeek in order to assist with language translation and polishing of the manuscript text. After using this tool, we reviewed and edited the content as needed and take full responsibility for the content of the published article.

## CRediT authorship contribution statement

**Changquan Wang:** Writing – review & editing, Writing – original draft, Methodology, Funding acquisition, Conceptualization. **Bin Chen:** Validation, Supervision, Software, Formal analysis. **Jing Hong:** Visualization, Software, Data curation. **Jiwen Xu:** Writing – review & editing, Validation, Supervision, Methodology.

## Ethical Statement

This study was approved by the Ruijin Hospital Luwan Branch Ethics Committee, Shanghai JiaoTong University School of Medicine. The patient and his legal guardians provided written informed consent before surgery.

## Funding

This work was funded by by the Youth Fund of the Health Commission of Huangpu District, Shanghai (HLQ202203); the Outstanding Youth Talent Training Program of Ruijin Hospital Luwan Branch, Shanghai Jiao Tong University School of Medicine (YQ202313).

## Declaration of competing interest

The authors declare that they have no known competing financial interests or personal relationships that could have appeared to influence the work reported in this paper.

## References

[1] Bassoe P., Nuzum F. (1915). Report of a case of central and peripheral neurofibromatosis. J. Nerv. Ment. Dis..

[2] Roux A., Zanello M., Mancusi R.L., MEH Still, Nascimento F.A., Tauziede-Espariat A. (2021). Meningioangiomatosis: multimodal analysis and insights from a systematic review. Neurology.

[3] Charan B.D., Goel V., Das S., Jain S., Garg A., LJD Sebastian (2025). Meningioangiomatosis with variable imaging feature: rare cause of drug refractory epilepsy. Asian J. Neurosurg..

[4] Yan C.G., Wang X.D., Zuo X.N., Zang Y.F. (2016). DPABI: data processing & analysis for (resting-state) brain imaging. Neuroinformatics.

[5] Alim-Marvasti A., Romagnoli G., Dahele K. (2022). Probabilistic landscape of seizure semiology localizing values. Brain Commun..

[6] Bartolomei F. (2024). The epileptogenic network concept: applications in the SEEG exploration of lesional focal epilepsies. Neurophysiol. Clin..

[7] Ikemoto S., von Ellenrieder N., Gotman J. (2022). Electroencephalography-functional magnetic resonance imaging of epileptiform discharges: noninvasive investigation of the whole brain. Epilepsia.

[8] Latini F., Hjortberg M., Aldskogius H., Ryttlefors M. (2015). The classical pathways of occipital lobe epileptic propagation revised in the light of white matter dissection. Behav. Neurol..

[9] Dimova P.S., Milenova Y., Stoyanova D., Karazapryanov P., Minkin K., Gabrovski K. (2025). Localizing value of ictal hypersalivation/sialorrhea and ictal spitting: a systematic review. Epileptic Disord.

[10] Loddenkemper T., Kotagal P. (2005). Lateralizing signs during seizures in focal epilepsy. Epilepsy Behav..

[11] Boerwinkle V.L., Cediel E.G., Mirea L. (2019). Network-targeted approach and postoperative resting-state functional magnetic resonance imaging are associated with seizure outcome. Ann. Neurol..

[12] Lee D.A., Lee H.J., Kim H.C., Park K.M. (2021). Alterations of structural connectivity and structural co-variance network in focal cortical dysplasia. BMC Neurol..

[13] Li C., Su H., Liu Y. (2025). Predicting surgical outcome in patients with drug-resistant epilepsy using autoregressive connectivity and virtual resection. IEEE J. Biomed Health Inform..

[14] Tomkinson C., Lu J.Q. (2018). Meningioangiomatosis: a review of the variable manifestations and complex pathophysiology. J. Neurol. Sci..

[15] Perry A., Kurtkaya-Yapicier O., Scheithauer B.W. (2005). Insights into meningioangiomatosis with and without meningioma: a clinicopathologic and genetic series of 24 cases with review of the literature. Brain Pathol..

[16] Goates J.J., Dickson D.W., Horoupian D.S. (1991). Meningioangiomatosis: an immunocytochemical study. Acta Neuropathol..

[17] Arcos A., Serramito R., Santín J.M. (2010). Meningioangiomatosis: clinical-radiological features and surgical outcome. Neurocirugia.

[18] Yao Z., Wang Y., Zee C., Feng X., Sun H. (2009). Computed tomography and magnetic resonance appearance of sporadic meningioangiomatosis correlated with pathological findings. J. Comput. Assist. Tomogr..

[19] Lemoine É., Jemel M., Xu A.Q. (2025). Prognostic value of interictal epileptiform discharges on routine EEG in adults with epilepsy. Epilepsia.

