# Supplementary material for: Network reprogramming after resection of occipital meningioangiomatosis: Evidence from multimodal localization and longitudinal fMRI
Source: Epilepsy Behav Rep. 2026 May 19;34:100874. doi: 10.1016/j.ebr.2026.100874 (PMC13266237; doi:10.1016/j.ebr.2026.100874)
Supplement: Supplementary file 1 — Supplementary material [file mmc1.docx]

**Supplementary Figure 1.** Long-term Video-EEG: One clinical seizure and three EEGs were recorded.

The ictal EEG and Electrographic seizures shown as a continuous recording, was obtained with electrodes arranged in the standardized 32-channel 10-20 system. SP1 refers to the left sphenoidal electrode, and SP2 to the right.

**Interictal EEG:**

Bilateral occipital sharp and slow wave discharges of medium amplitude.


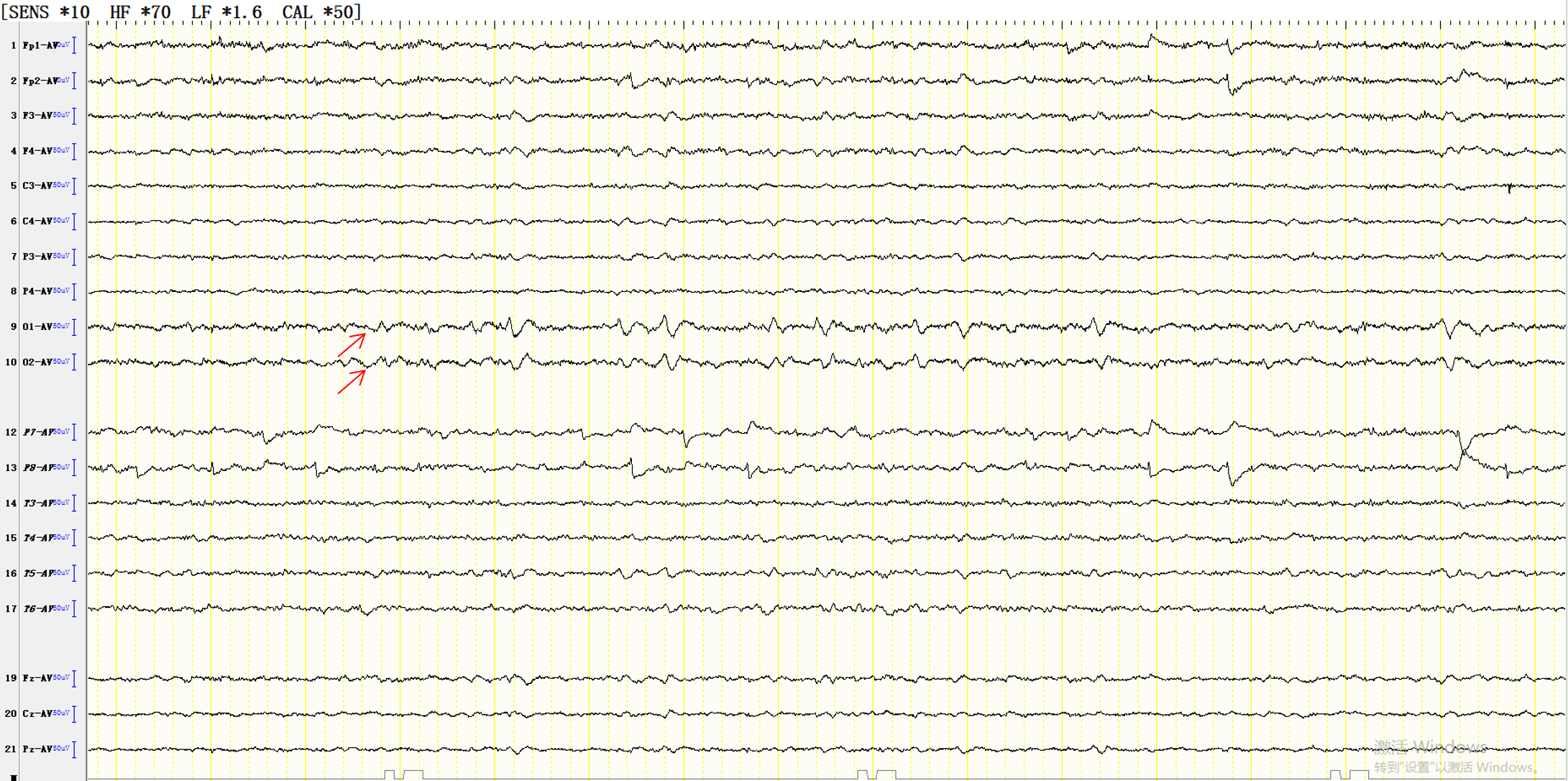


**Interictal EEG:** Synchronous spikes and sharp waves were recorded from the bilateral sphenoidal electrodes and temporal regions.


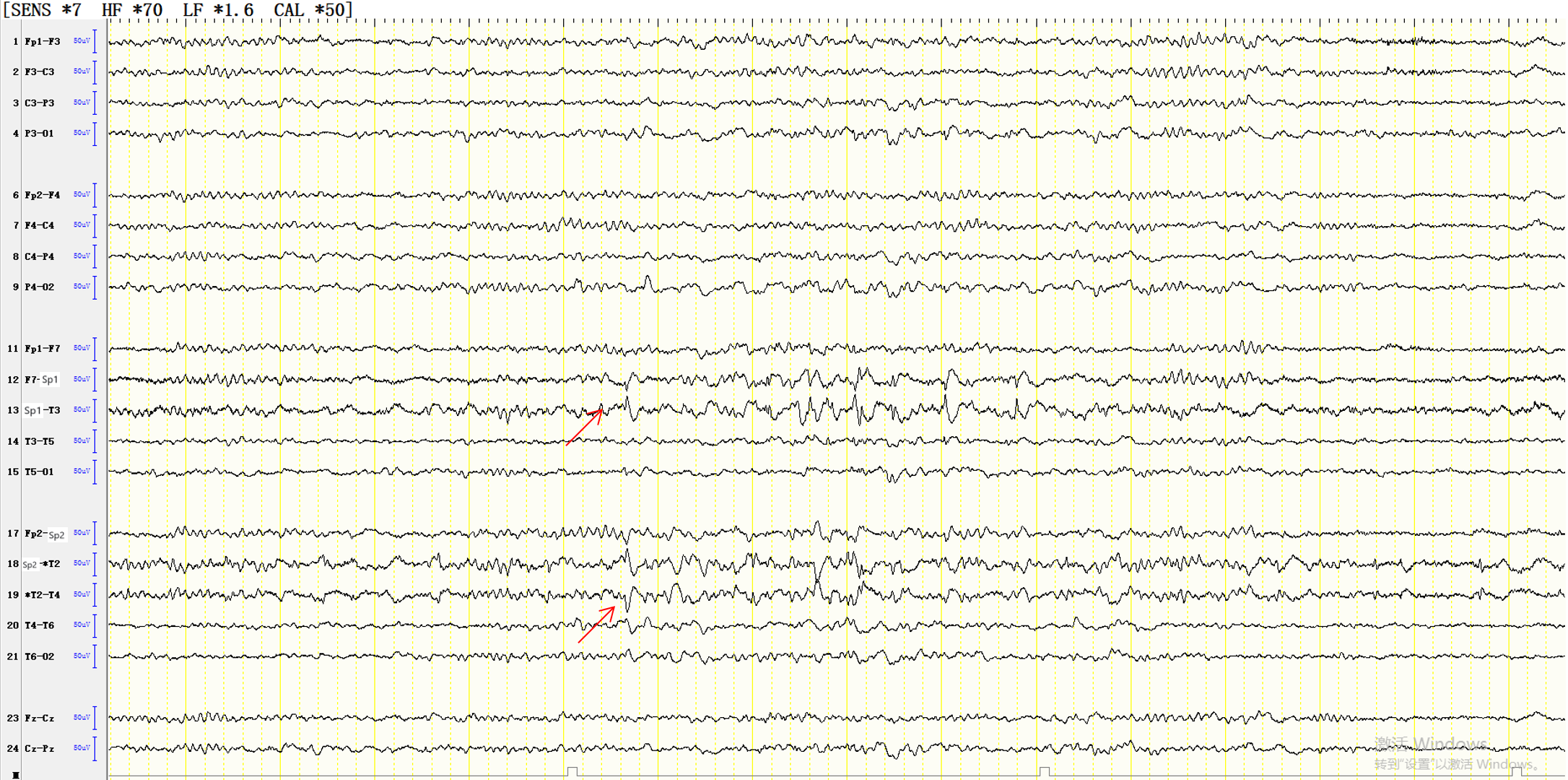


**Interictal EEG: Spikes and sharp waves in the bilateral sphenoidal electrodes and temporal regions, predominant on the left side.**


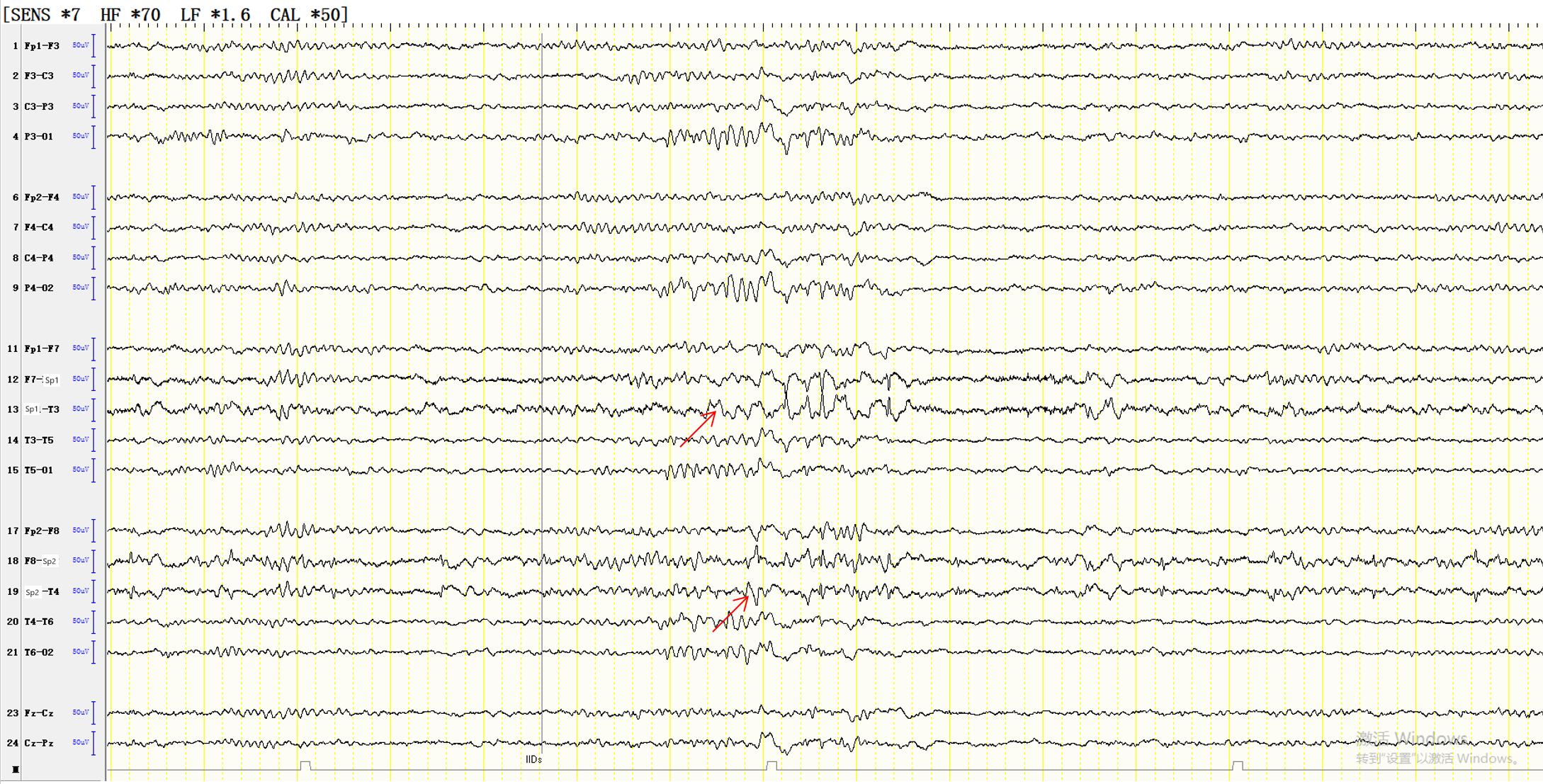


**Interictal EEG:** **Spikes and sharp waves in the bilateral sphenoidal electrodes and temporal regions, predominant on the right side.**


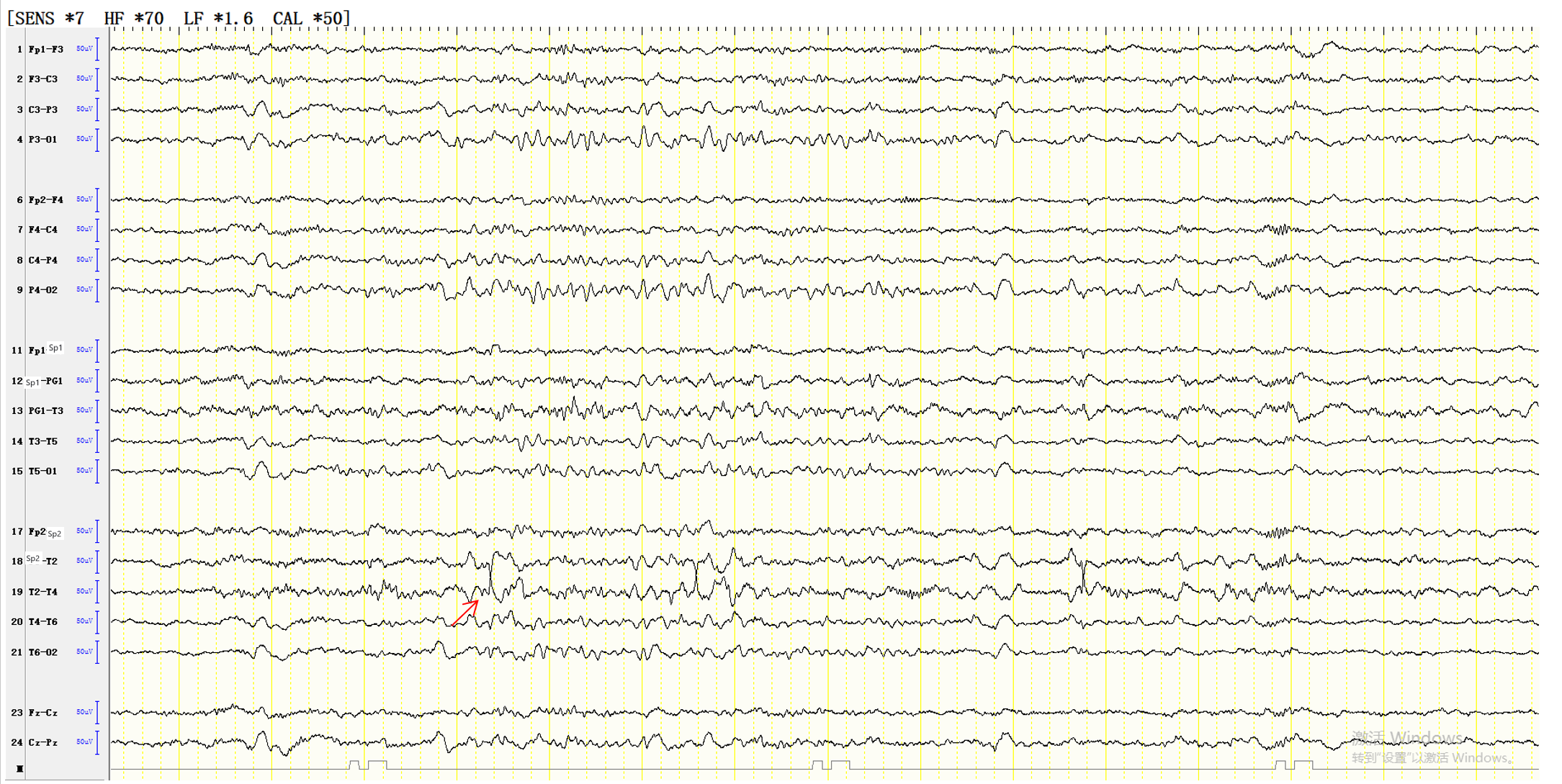


**Ictal EEG (Clinical Seizure):**

Onset with rhythmic, low-amplitude sharp activity in the right parietal–occipital region. Rapid anterior propagation evolved into rhythmic low-to-medium-amplitude sharp–slow complexes in right sphenoidal, temporal, frontopolar, and frontal regions, eventually bilateral/diffuse. The following four figures are consecutive screenshots.


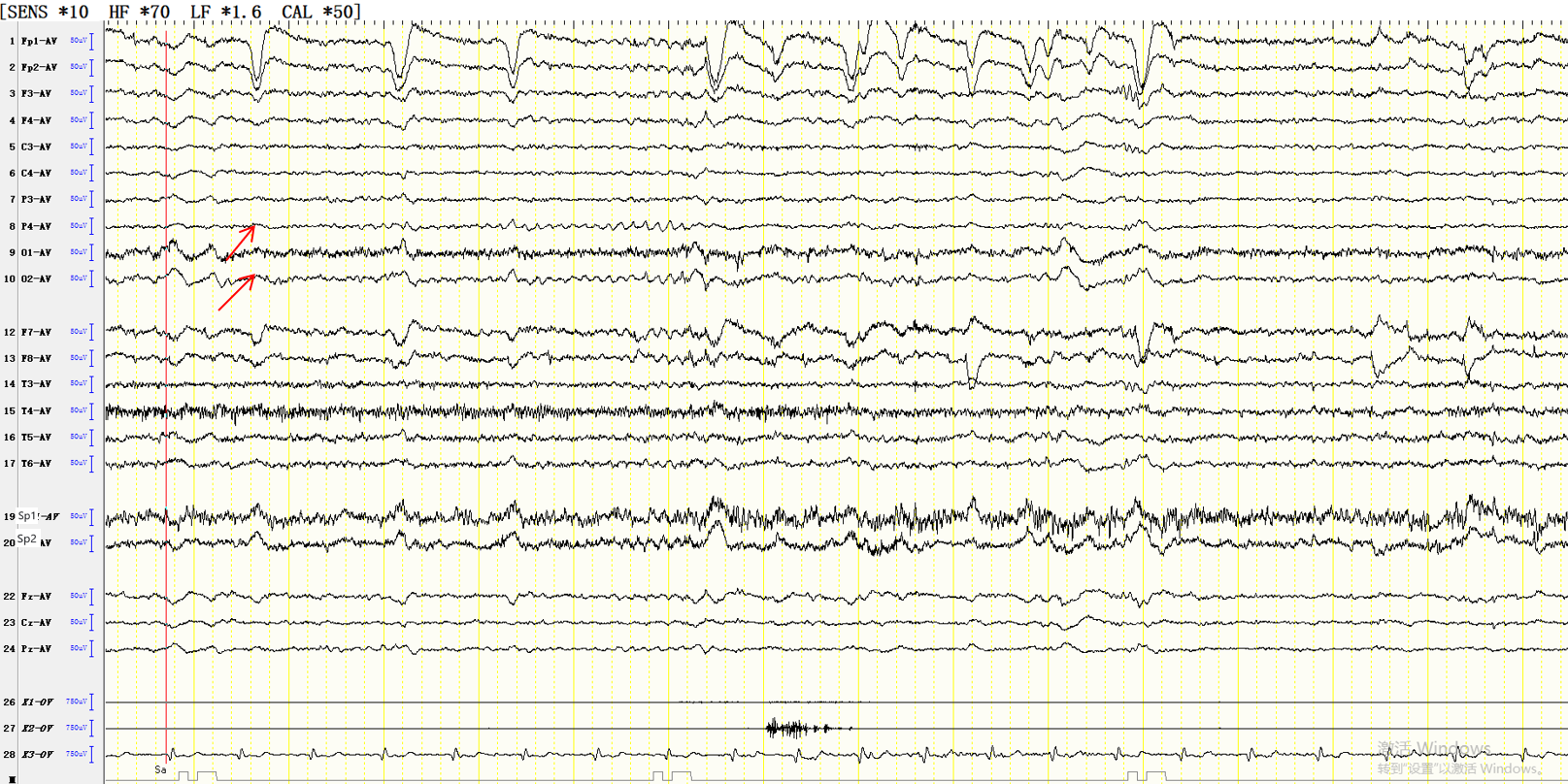

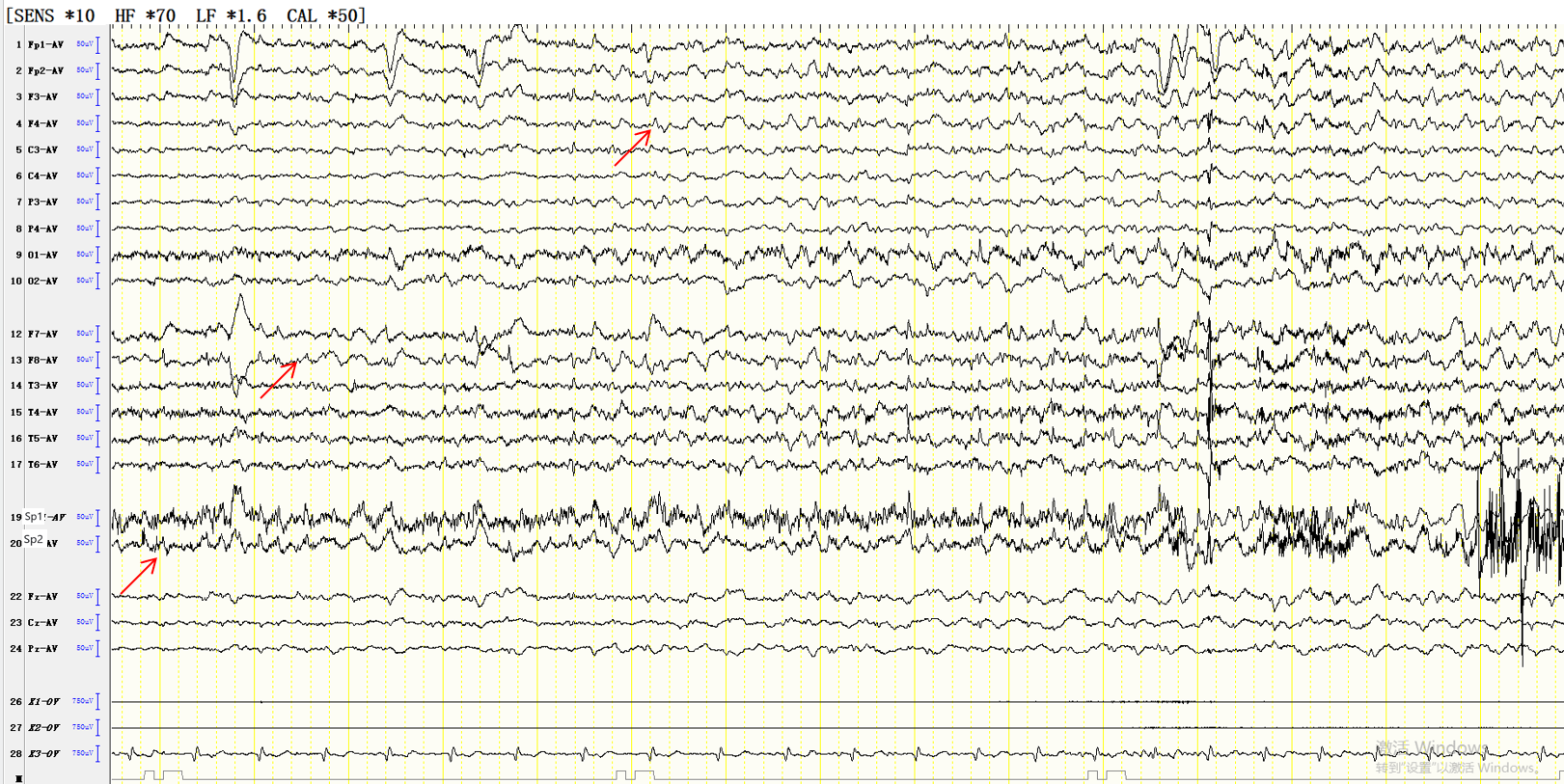

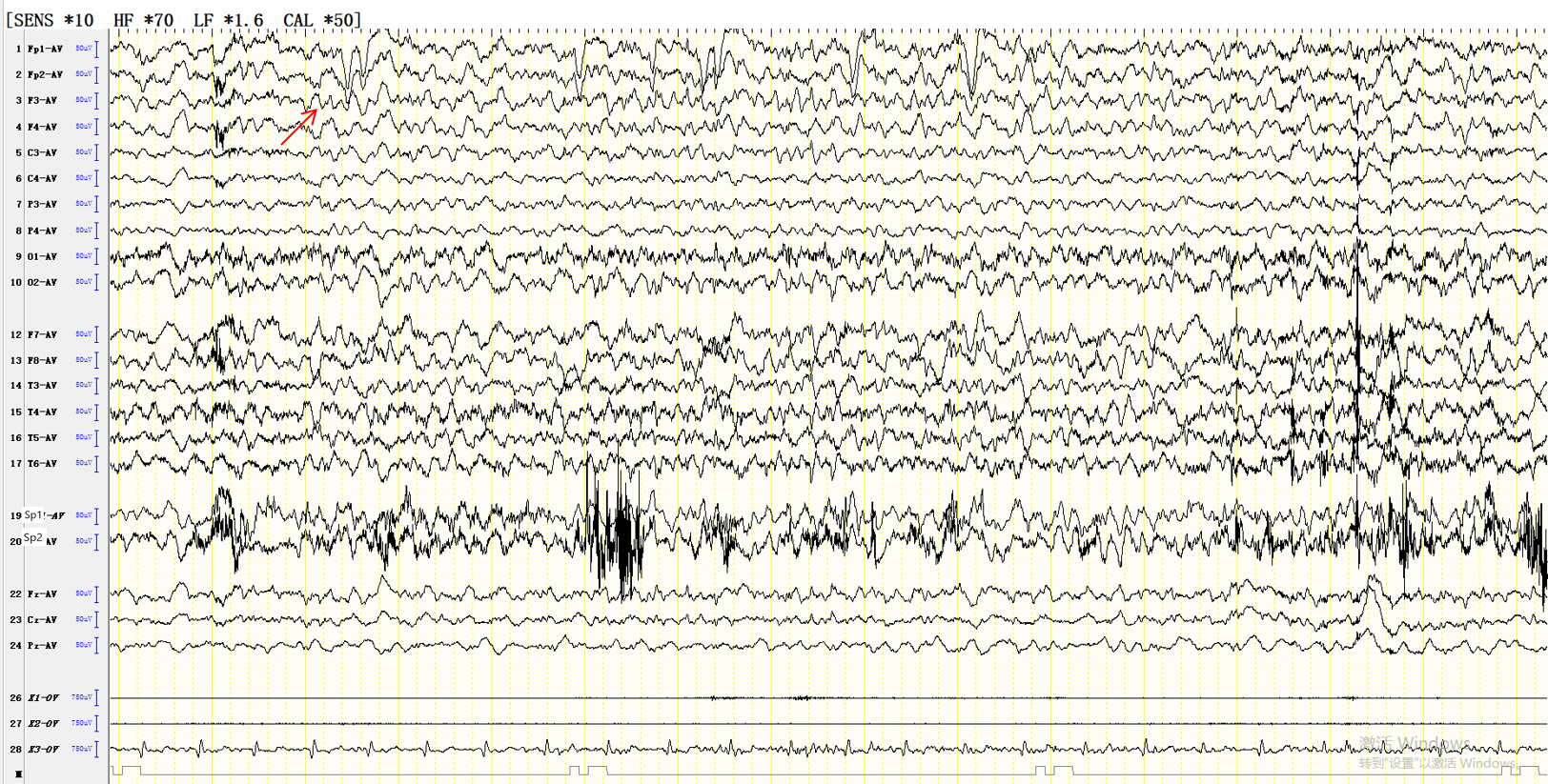

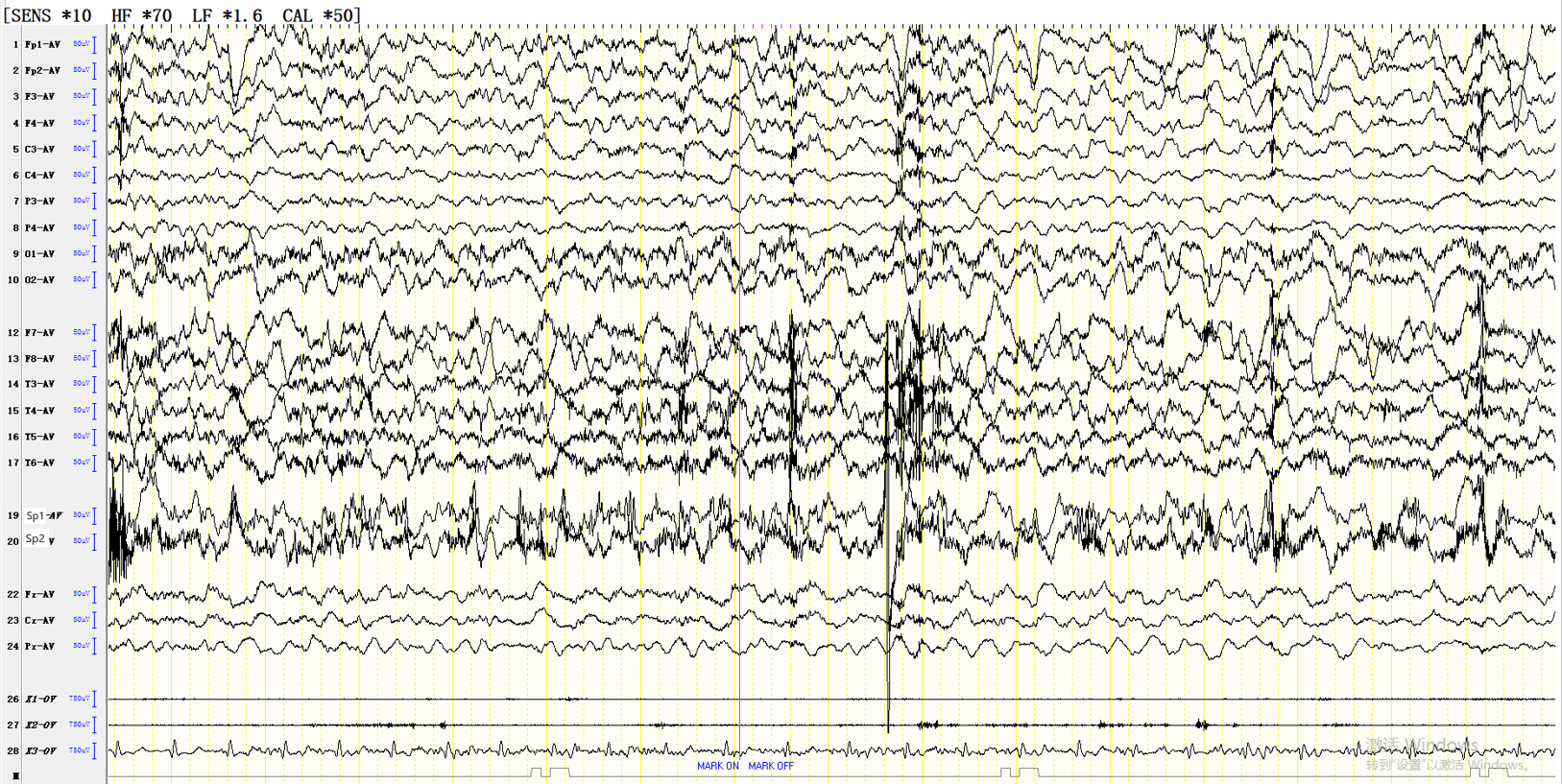


**Electrographic seizures (n=3; all with coughing: occurring once during a seizure and twice at its termination).**

Onsets commonly emphasized left frontal/central/parietal channels with low-amplitude slow waves, followed by bilateral temporal spike recruitment (right-predominant), then spread to frontal/central/temporal regions and diffuse involvement (~70 s). The following four figures are consecutive screenshots.


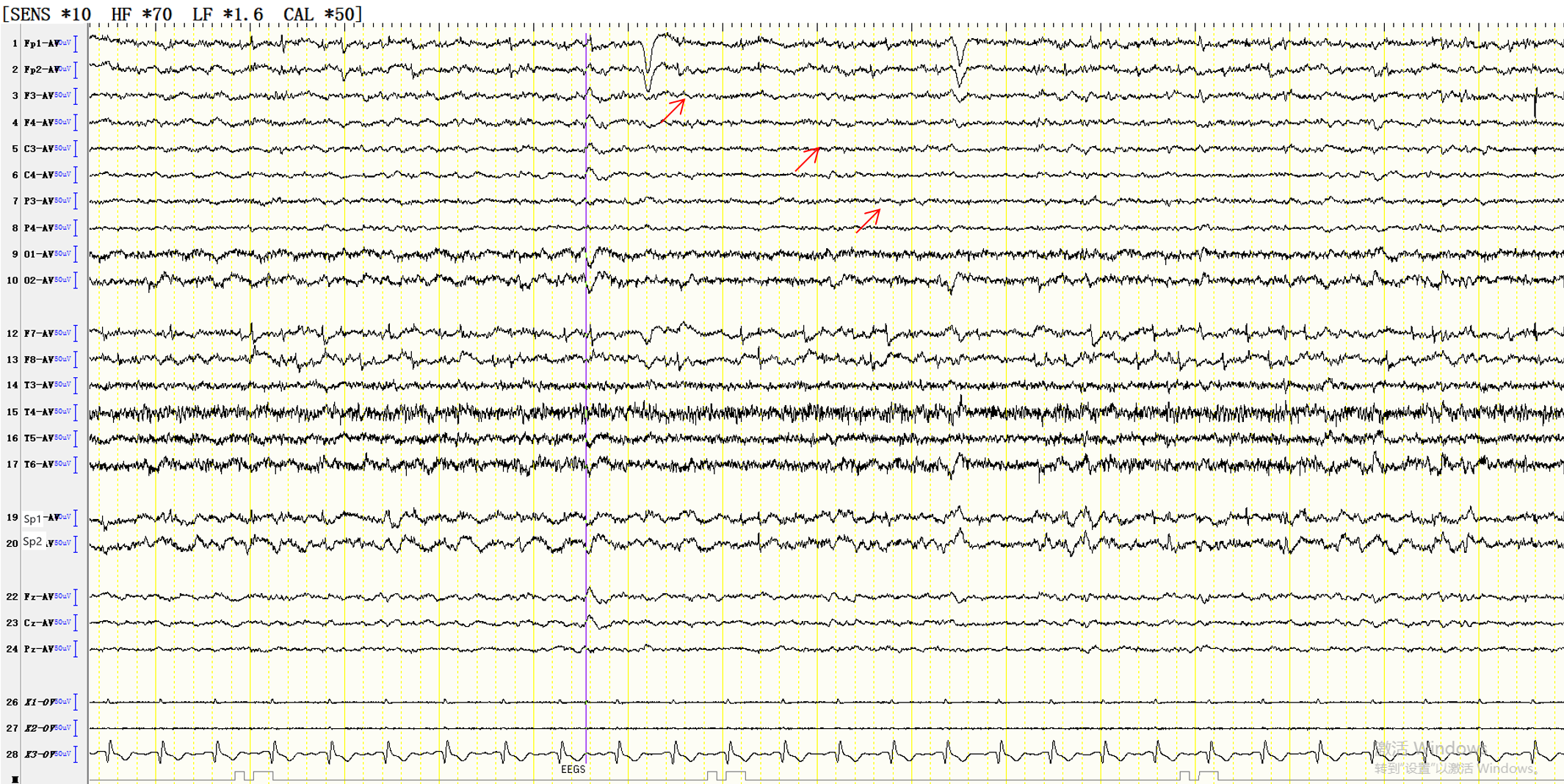


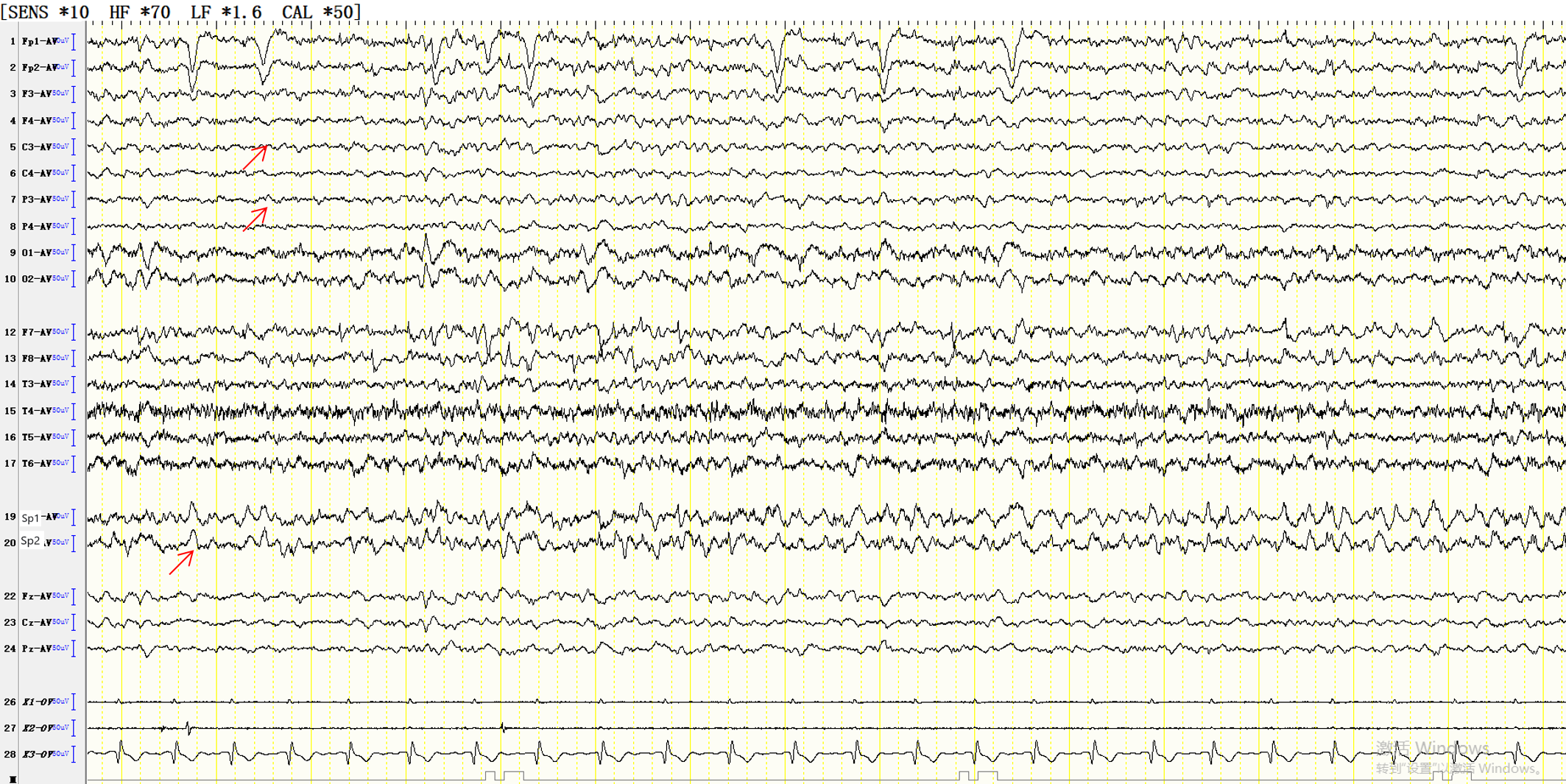


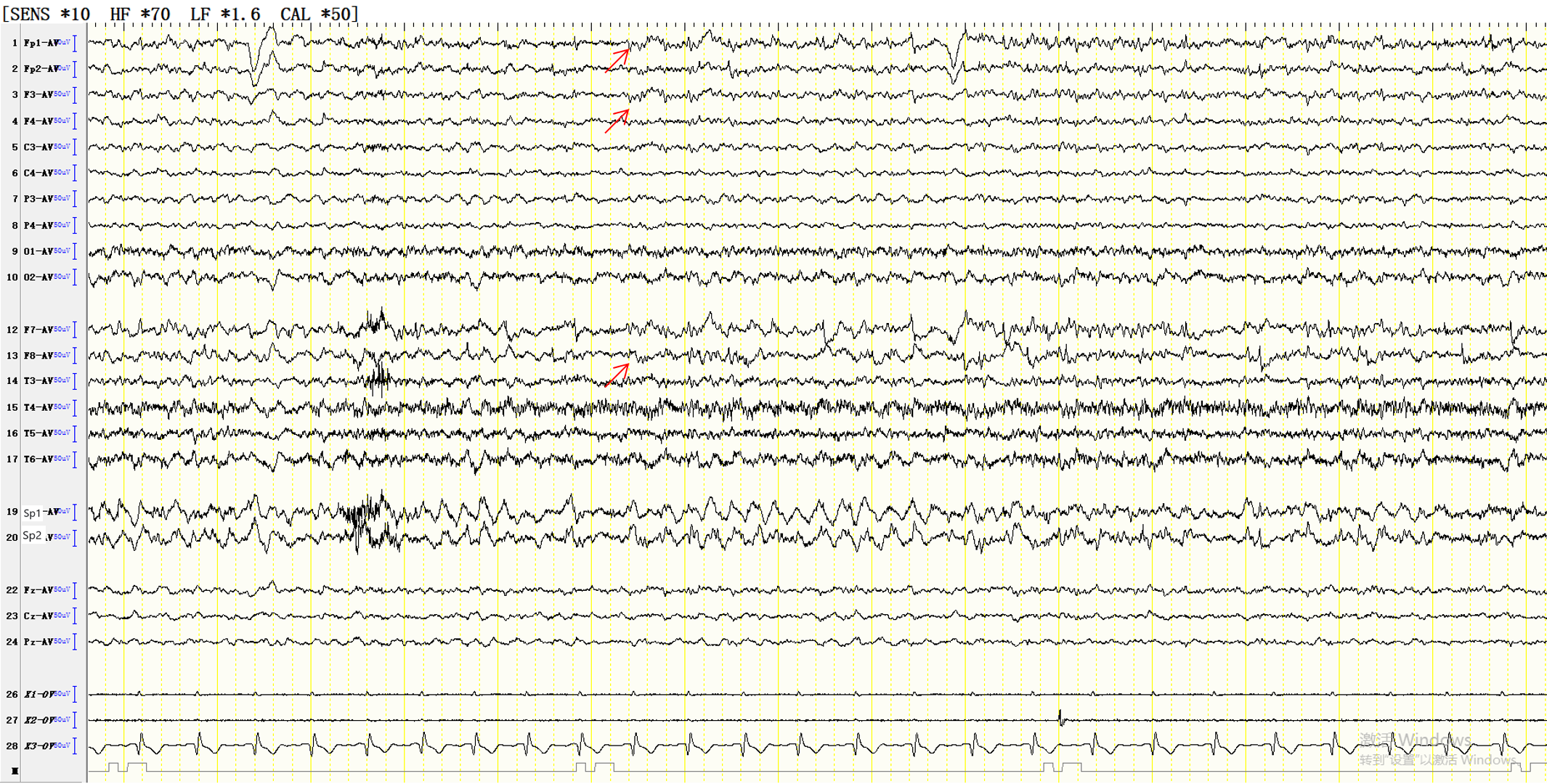


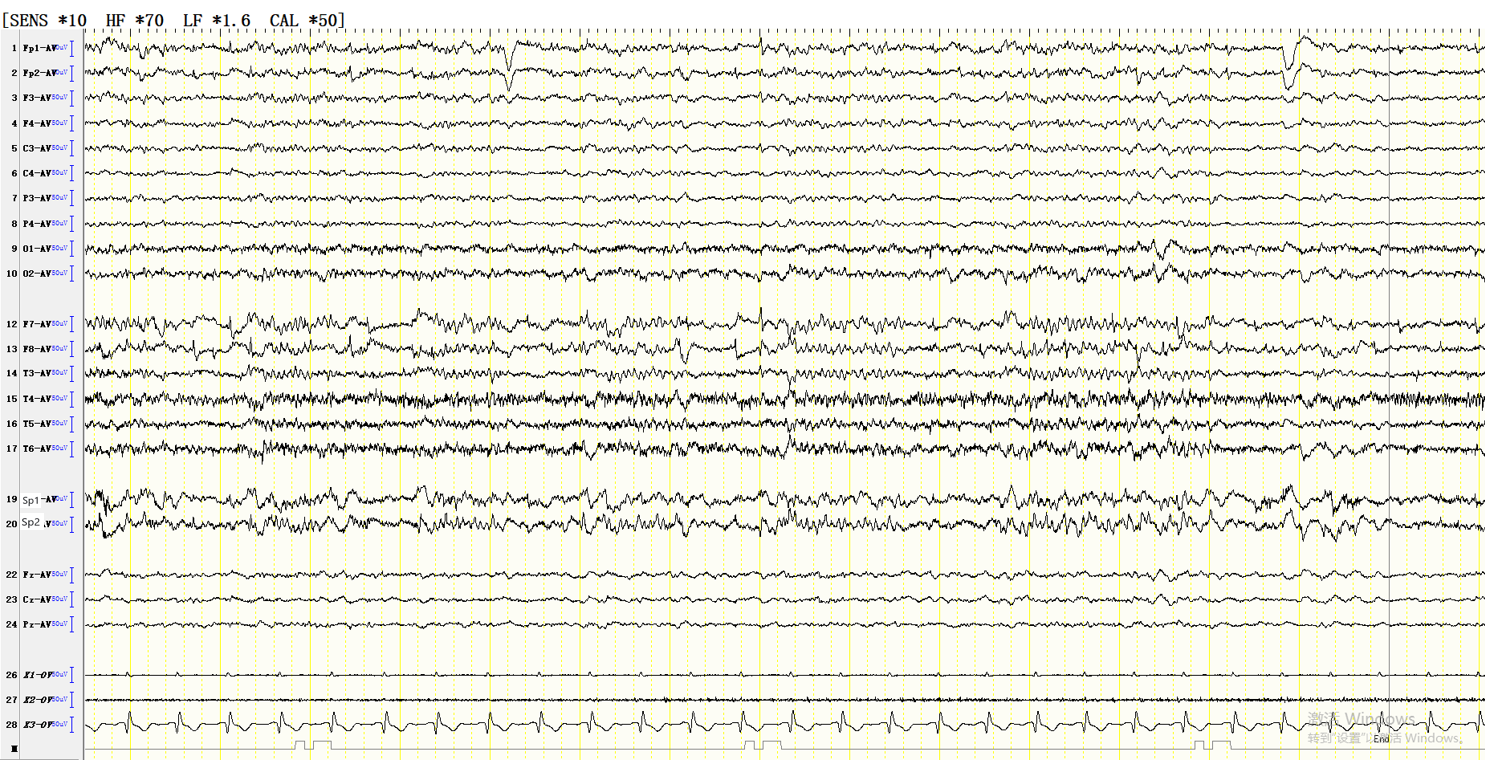


**Supplementary Figure 2.** Postoperative EEG at three months. Background activity showed mild slowing over the right hemisphere. No typical epileptiform discharges were identified during wakefulness or sleep.


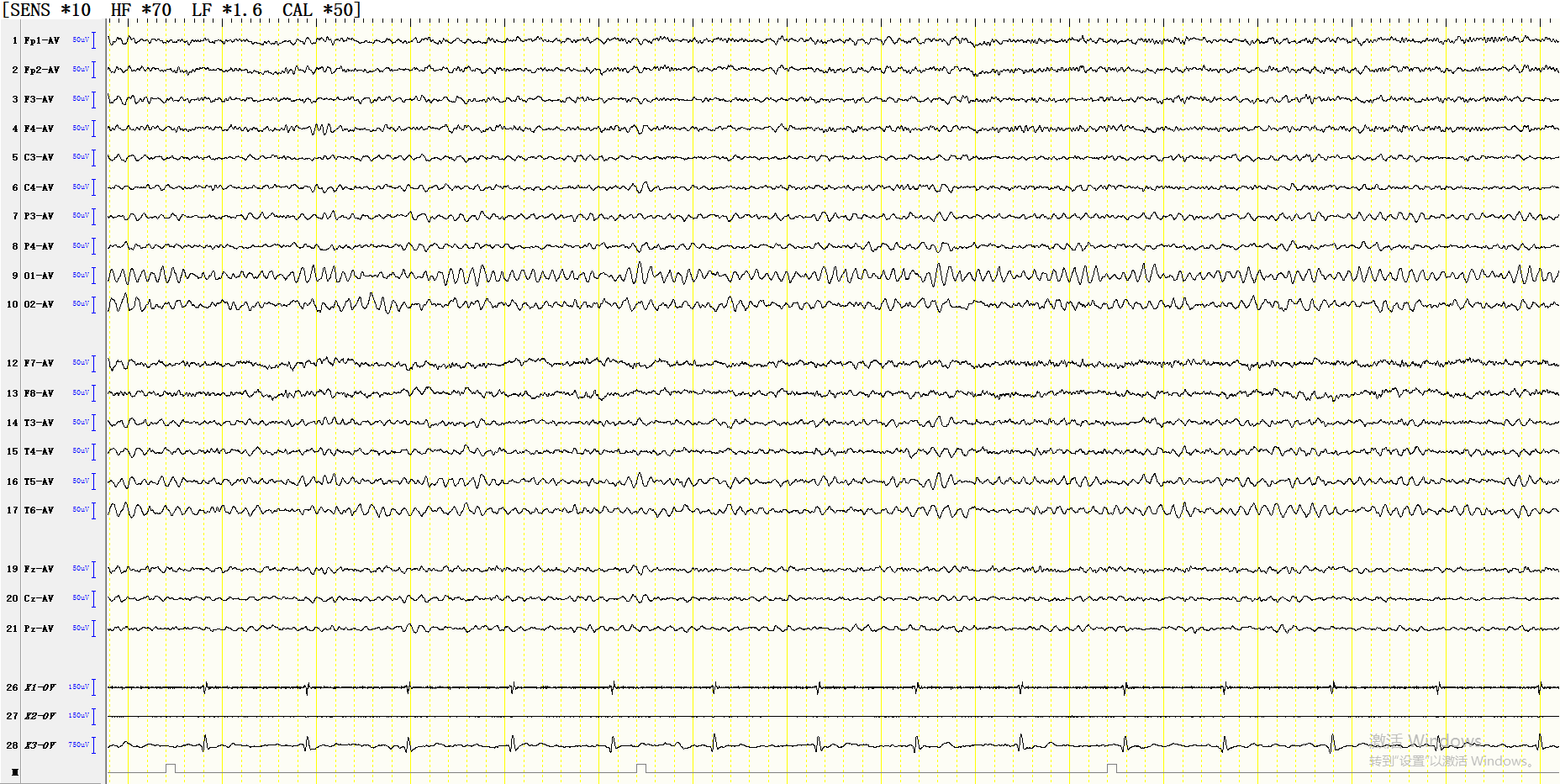


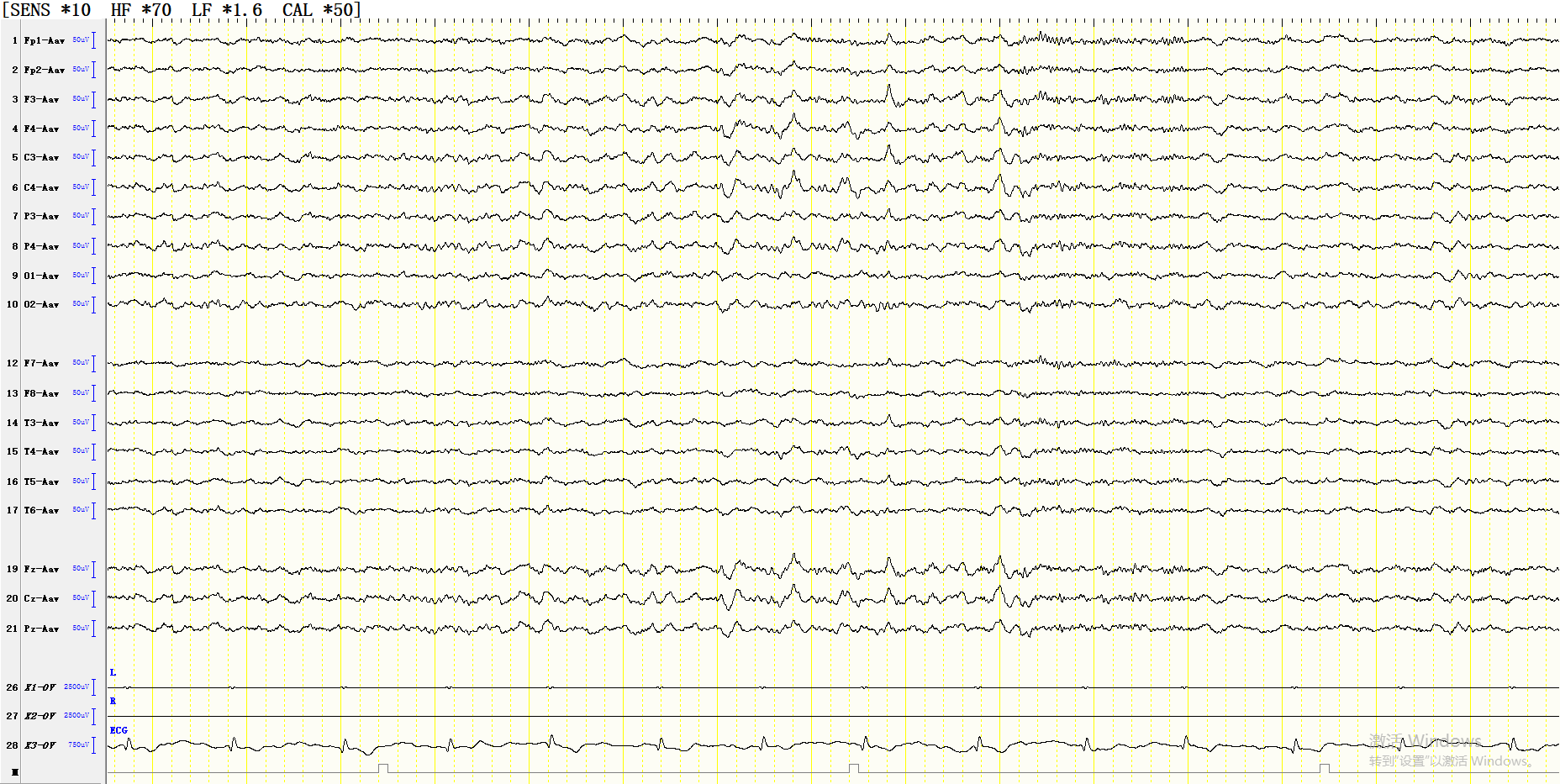


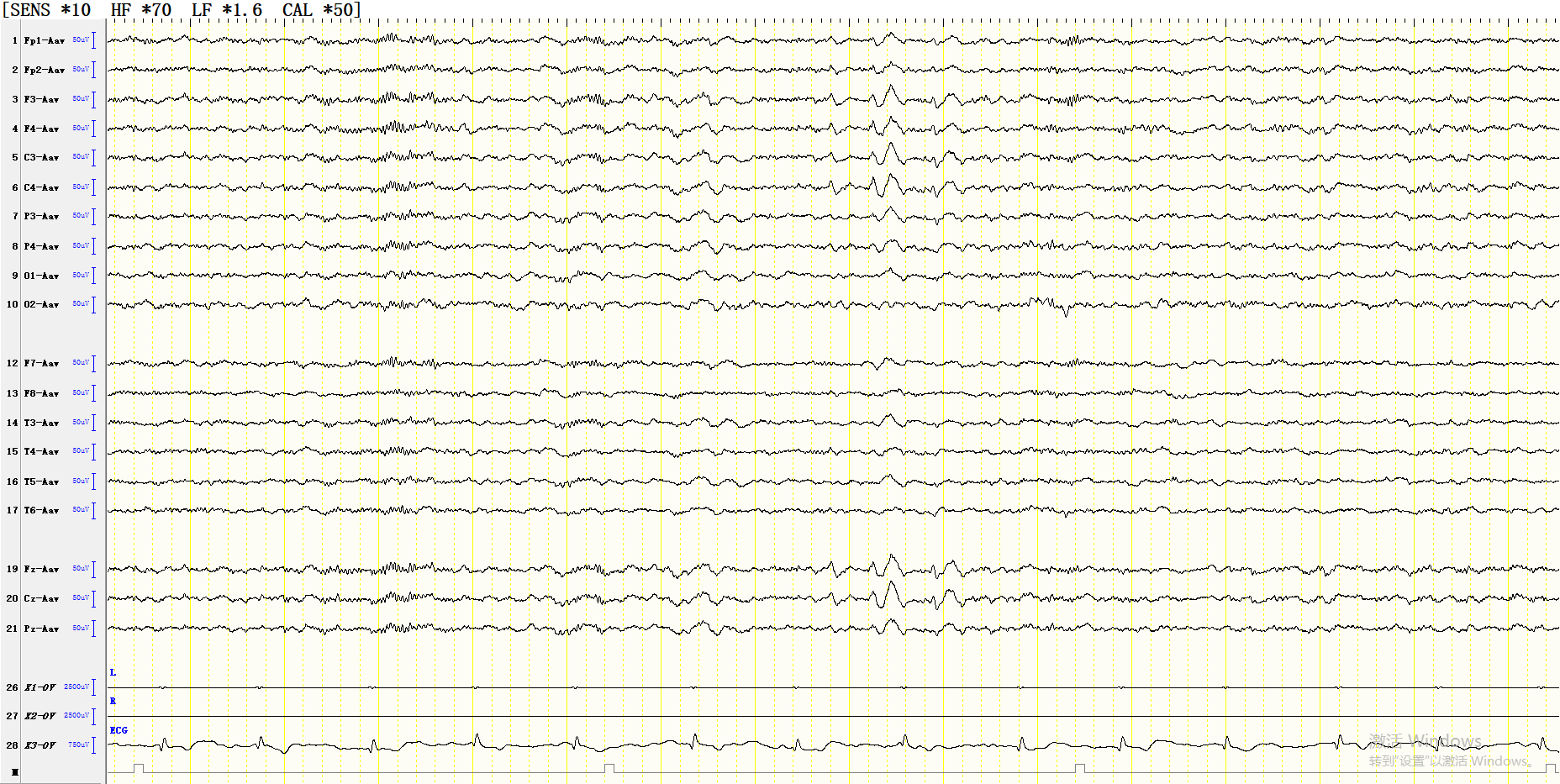


**Supplementary Table 1.** List of abbreviations used in the manuscript.

| Abbreviation | Full Term |
| --- | --- |
| CSF | Cerebrospinal fluid |
| CT | Computed tomography |
| DC | Degree centrality |
| DPABI | Data Processing & Analysis for Brain Imaging |
| DMN | Default mode network |
| EEG | Electroencephalography |
| fALFF | Fractional amplitude of low-frequency fluctuation |
| FC | Functional connectivity |
| FDG-PET | Fluorodeoxyglucose positron emission tomography |
| FLAIR | Fluid-attenuated inversion recovery |
| fMRI | Functional magnetic resonance imaging |
| GM | Gray matter |
| MA | Meningioangiomatosis |
| MRI | Magnetic resonance imaging |
| PET | Positron emission tomography |
| ReHo | Regional homogeneity |
| ROI | Region of interest |
| rs-fMRI | Resting-state functional magnetic resonance imaging |
| SMA | Supplementary motor area |
| WM | White matter |
